# Safety and acceptability of *Lactobacillus reuteri* DSM 17938 and *Bifidobacterium longum* subspecies *infantis* 35624 in Bangladeshi infants: a phase I randomized clinical trial

**DOI:** 10.1186/s12906-016-1016-1

**Published:** 2016-02-02

**Authors:** Yana Emmy Hoy-Schulz, Kaniz Jannat, Thomas Roberts, Saira Husain Zaidi, Leanne Unicomb, Stephen Luby, Julie Parsonnet

**Affiliations:** 1 Department of Medicine, Division of Infectious Diseases and Geographic Medicine, Stanford University School of Medicine, 300 Pasteur Drive, Grant S-107, Stanford, CA USA; 2 International Center for Diarrheal Disease Research, Bangladesh (ICDDR,B), Dhaka, Bangladesh; 3 School of Public Health, University of California, Berkeley, Berkeley, CA USA

**Keywords:** Probiotics, Safety, Infants, *Lactobacillus*, *Bifidobacterium*

## Abstract

**Background:**

Probiotics have rarely been studied in young healthy infants from low-income countries. This phase I study investigated the safety and acceptability of two probiotics in Bangladesh.

**Methods:**

Healthy infants aged four to twelve weeks from urban slums in Bangladesh were randomized to one of three different intervention dosing arms (daily, weekly, biweekly – once every two weeks) of *Lactobacillus reuteri* DSM 17938 and *Bifidobacterium longum* subspecies *infantis* 35624 over one month or to a fourth arm that received no probiotics. All subjects were followed for two additional months. Reported gastrointestinal and respiratory symptoms as well as breastfeeding rates, hospitalizations, differential withdrawals, and caretakers’ perception of probiotic use were compared among arms.

**Results:**

In total, 160 infants were randomized (40 to each arm) with 137 (Daily *n* = 35, Weekly *n* = 35, Biweekly *n* = 35, Control *n* = 32) followed up for a median of twelve weeks; 113 completed the study. Illness and breastfeeding rates were similar across all arms. Ten hospitalizations unrelated to probiotic use occurred. Forty eight percent of the caretakers of infants in intervention arms believed that probiotics improved their baby’s health.

**Conclusions:**

These two commonly used probiotics appeared safe and well-accepted by Bangladeshi families.

**Trial registration:**

ClinicalTrials.gov NCT01899378. Registered July 10, 2013.

## Background

Probiotics are living microbial organisms that when administered in adequate amounts confer health benefits to the host [1]. Although a number of probiotic strains have been shown to be safe and to improve health by a variety of mechanisms [2, 3], few studies have assessed probiotics in healthy young infants in low-income countries. Gut microbiota of children in low-income countries differ from those in wealthier nations [4]. Gastrointestinal pathogens and diarrheal disease are more common in children in low-income countries, as is environmental enteropathy, a condition of increased intestinal permeability and decreased nutrient absorption [5]. We and others speculate that probiotics could modify gut microbiota, enhance gut immunity, and decrease gastrointestinal disease risk, especially if administered early in life [6]. Furthermore, probiotics administered early in life may have the greatest potential to achieve long-term colonization and immunologic benefits, before infants have begun to receive complementary foods and their microbiota shift to a more mature assemblage. However, it is possible that probiotics may induce adverse events in young infants with dysbiosis, reduced intestinal integrity, and decreased immunity [7, 8]. Therefore, determining safety in this infant population is crucial.

The choice of probiotics in this study was based on a literature review and strong safety data in infants. *Lactobacillus reuteri* DSM 17938 (parent strain *L. reuteri* ATCC 55730) [9] has been safely used in infants [10] and adults in the US and Europe [11] and recently in adults in the Peruvian Amazon [12] and has been reported to prevent or reduce diarrhea and gastrointestinal and respiratory infections [13–15], reduce pathogen colonization and alter microbiota composition [16, 17], reduce infant colic and crying time [18–20], suppress *Helicobacter pylori* and gastric symptoms [21], relieve constipation [22], control reflux and abdominal pain [23], and improve infant weight gain [24]. *Bifidobacterium longum* subspecies *infantis* is commonly found in both breast milk [25] and healthy infant stools [4] and is generally recognized as safe [26].

With the ultimate goal of evaluating the efficacy of probiotics to improve health in children in Bangladesh, the objectives of this report are to assess the safety and acceptability of three different regimens of *Lactobacillus reuteri* DSM 17938 and *Bifidobacterium longum* subspecies *infantis* 35624 given over one month to very young healthy infants.

## Methods

### Study population

Infants were recruited from three vaccination clinics near the International Center for Diarrheal Disease Research, Bangladesh (ICDDR,B) in Dhaka between October 2013 and April 2014. Inclusion criteria were: a) four to twelve weeks of age; b) no birth defects, history of hospitalization, or ongoing acute or chronic illness; c) no current antibiotic or probiotic use; d) weight within three standard deviations of the norm; and e) local residence for next four months. To select for infants who were more likely to be affected by environmental enteropathy, gastrointestinal infections, malnutrition, and stunting, children from lower socioeconomic status communities from households that shared a kitchen, water source, latrine, or courtyard with at least one other household were recruited. No restrictions on the diet of participating infants were made during the study. Parents or guardians provided written informed consent. The study was approved by the institutional review boards at both ICCDR,B (Protocol ID 13022) and Stanford University (Protocol ID 25487) and was registered on ClinicalTrials.gov (NCT01899378).

For the primary outcome of this study, proportion of days with symptoms, to detect a difference in change of 0.15 between the two groups with a sigma of 0.18 and an alpha of 0.05 and greater than 80 % power, 25 infants were needed per group. Twenty five infants in each group would allow detection of almost a two-fold difference (74 % vs. 35 %) between arms with 80 % power for dichotomous variables such as breastfeeding rates. Because the study population was transient, high levels of drop-out and loss to follow-up were expected. A similar study in this population had a 38 % drop-out rate; therefore 40 infants were enrolled per arm in order achieve a final sample size of at least 25 infants per arm [27].

### Study design

**Fig. 1:**
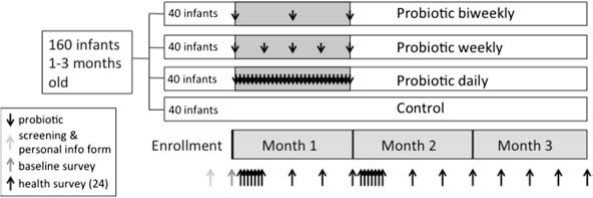
Study design and sampling scheme

The study design was multi-arm parallel where infants were randomized in equal numbers to one of four arms – a control arm (observation only) or to one of three intervention arms of *L. reuteri* DSM 17938 (10^8^ colony forming units (CFU)) and *B. longum* subspecies *infantis* 35624 (10^9^ CFU): daily dosing (29 doses overall), weekly dosing (five doses), or every two week dosing (three doses). Block randomization using a computerized random number generator and block sizes of twenty was used to account for seasonal differences. The primary researcher generated the sequence and the field research officer in charge of coordinating enrollment was blinded to block sizes. Enrollment was conducted by multiple field team members simultaneously. After screening and obtaining consent for an infant, the field team member contacted the field research officer for the next enrollment identification number to be assigned. Post-intervention follow-up occurred for two months (Fig. 1). Data collection ended in July 2014 when the final participating infant completed follow-up.

### Intervention

*L. reuteri* DSM 17938 (BioGaia, Sweden), in liquid drops, was stored and transported to the field at 4–8 °C; each dose was five drops. *B. longum* subspecies *infantis* 35624 (Proctor and Gamble), a powder-containing capsule, was stored and transported at ambient temperature. Immediately before administration the capsule was opened and the power was mixed with < 500ul sterile water to create a liquid suspension. Both probiotics were fed to infants by study staff using sterile Pasteur pipets in participating infants’ homes. Mothers were encouraged to breastfeed infants after each probiotic administration to ensure that the probiotic was swallowed. Infants were monitored by study staff for at least 30 min for immediate adverse reactions.

### Data collection

Demographic and socioeconomic data were collected at enrollment. Health information, including gastrointestinal and respiratory symptoms as well as breastfeeding practices, was collected from caregivers on each of the seven days after the first and last probiotic doses and weekly at all other times covering the interval since last visit (Fig. 1). Exclusive breastfeeding was defined as the reported receipt of only breast milk in the prior 24 h [28]. At the end of the study, data were collected regarding the caretakers’ perceptions of probiotics. All survey data were collected electronically by study staff with Open Data Kit (ODK) software (https://opendatakit.org/) on portable tablets [29]. All infant hospitalizations were immediately reported to and reviewed by the Data Safety Monitoring Board (DSMB) at the ICCDR,B. Infants who were hospitalized continued in the study after they were released from the hospital unless they were withdrawn from the study by their parents.

### Statistical analysis

Intent-to-treat analysis of those who initiated the study after randomization was performed; infants for whom no follow-up data were available were not included. Baseline demographic characteristics of infants and their households were compared among arms using the Kruskal-Wallis or chi-squared test. The primary outcomes of gastrointestinal and respiratory symptoms per infant (days with symptoms/total follow-up days) were compared with the Kruskal-Wallis test and a test for trend. Withdrawals, symptoms and monthly household income within and between the arms were compared using two-way ANOVA or chi-squared tests. Rates of exclusive breastfeeding among arms were compared using difference in proportion or chi-squared tests.

There were concerns by the ICDDR,B review committee that oral administration of probiotics could affect breastfeeding rates. Therefore, a stopping rule was implemented that if probiotics caused a reduction in breastfeeding, the trial would be stopped. At midline, interim analysis on breastfeeding data only was conducted and reported to the Data Safety Monitoring Board.

## Results

**Fig. 2:**
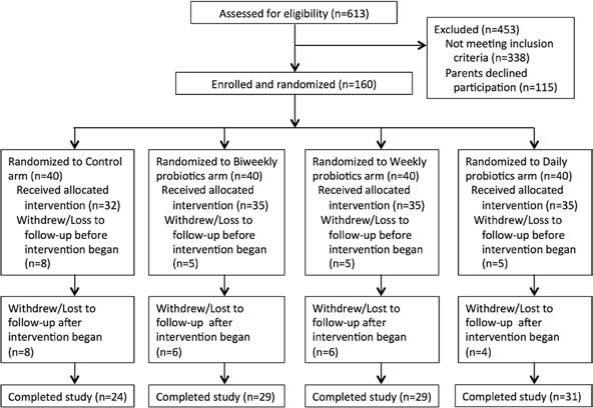
Flow-chart of study screening, enrollment, and retention

A total of 613 infants were screened; 275 were considered eligible (45 %) and 160 were enrolled (26 %), with 40 randomized to each study arm (Fig. 2). The most common reasons for ineligibility were moving outside of Dhaka (*n* = 97) or the infant was not currently healthy (*n* = 122). A total of 23 infants withdrew or were lost to follow-up after baseline data collection but before the intervention began, leaving 137 infants (86 %) contributing post-intervention data with a median follow-up of twelve weeks. These 137 infants were similar across arms (Table 1) although monthly household income was slightly lower in the weekly and daily treatment arms (*p* = 0.04). Of the 137 babies who contributed post-intervention data, 113 infants (82 %) completed the three months post-intervention of the study.

**Table 1:** Baseline characteristics of the study population

Characteristic		Control (*n* = 32)	Biweekly (*n* = 35)	Weekly (*n* = 35)	Daily (*n* = 35)
Age in weeks [mean (SD)]		7.7 (2.3)	8.1 (2.0)	8.3 (2.0)	8.2 (2.3)
Female infant [n, (%)]		18 (56)	16 (46)	16 (46)	18 (51)
Born by cesarean section [n (%)]		11 (34)	6 (17)	8 (23)	9 (26)
Previous antibiotic use [n (%)]		5 (17)	11 (31)	10 (31)	8 (24)
Bowel movements in past 24 h [mean (SD)]		2.25 (1.8)	2.7 (2.0)	2.5 (1.6)	3.2 (2.6)
Years of maternal education (formal schooling) [mean (SD)]		5.3 (3.0)	5.5 (3.2)	4.7 (3.3)	4.6 (2.6)
Household size [mean (SD)]		4.7 (1.6)	4.7 (1.8)	4.3 (1.3)	4.5 (2.0)
Household monthly income [n, (%)]					
	<$100	7 (22)	5 (14)	14 (40)	14 (40)
	$100-$150	16 (50)	11 (31)	11 (31)	10 (29)
	>$150	9 (28)	19 (54)	10 (29)	11 (31)

### Withdrawals

A total of 47 infants (29 %) withdrew, 23 before and 24 after initiation of the intervention (Fig. 2). The primary reason for withdrawal after the intervention was a move away from Dhaka (58 %), followed by the family was too busy (13 %), and the family perceived no benefit from study (13 %). Families in the control arm withdrew more frequently (40 %) than the biweekly (28 %), weekly (28 %), or daily arms (23 %); they also withdrew sooner. Within the lowest household income group (<$100 per month), 70 % in the control arm withdrew, compared to 40 %, 31 %, and 14 % in the biweekly, weekly and daily arms respectively (*p* = 0.04). At baseline 156 infants were being breastfed, three were already weaned, and one withdrew before any baseline information was collected.

### Safety

Cough and congestion were the most commonly reported symptoms (median 12 % and 14 % of follow-up days, respectively). Gastrointestinal symptoms were rare (Table 2). No differences were observed in percentage of follow-up time with diarrhea, watery or soft stool, vomiting, poor feeding, colic, cough, congestion, or difficulty breathing across arms (Table 2). Seventy percent of infants were being exclusive breastfed at eight weeks of age, while only 30 % were being exclusively breastfed at twenty weeks of age. No differences in exclusive breastfeeding rates were seen among study arms throughout the study. Eight infants (four from the biweekly arm, two from the weekly arm, two from the daily arm, and zero from the control arm) were hospitalized a total of ten times—six for pneumonia and four for diarrhea; all infants recovered fully. No hospitalization was temporally related to probiotic use or considered probiotic-related by the DSMB. Three infants had been weaned before enrollment; all three were hospitalized (50 % of hospitalizations). No allergic responses or other reactions were observed after probiotic administration.

**Table 2:** Median percent of follow-up time with symptoms per infant

Symptom [Median (SD)]	Control *n* = 32	Biweekly *n* = 35	Weekly *n* = 35	Daily *n* = 35	*p*-value	p-for trend
Diarrhea	0 (2.2)	0 (2.2)	0 (0.5)	0 (1.5)	0.7	0.9
Watery or soft stool	0 (3.8)	0 (7.3)	1.1 (5.5)	1.0 (5.6)	0.3	0.3
Vomiting	0 (3.2)	0 (4.8)	0 (2.9)	0 (2.8)	0.4	0.8
Poor feeding	0 (4.1)	3.3 (8.4)	1.1 (6.6)	2.1 (4.0)	0.8	0.5
Colic	0 (9.0)	0 (6.7)	1.1 (3.0)	1.1 (3.9)	0.5	0.4
Cough	10.0 (17.1)	12.3 (17.1)	14.2 (14.8)	12.8 (12.7)	0.5	0.7
Congestion	9.1 (18.2)	14.8 (17.2)	13.6 (13.0)	15.7 (11.1)	0.4	1.0
Difficulty breathing	0 (4.1)	0 (5.6)	0 (4.5)	0 (1.2)	0.2	0.3

Kruskal-Wallis Test, Test for Linear Trend

Significance for *p*-values was set at 0.05

### Perception of probiotics

When caretakers in the intervention arms were asked about probiotics, 48 % reported the probiotics improved the health of the baby, 6 % reported no benefit, and 46 % were undecided. This finding was similar across intervention arms (*p* = 0.54).

## Discussion

In this study, it was found that two probiotics were safe – did not cause sudden reactions, increase symptom rates, or diminish breastfeeding rates – and acceptable in infants younger than six months of age. No problems administering the probiotics were identified, with infants able to suck and swallow the formulations without difficulty or aversion. No differences in rates of any reported symptoms were observed among arms; additionally, no sudden adverse or allergic reactions were found after probiotic administration, and no hospitalizations were deemed related to probiotics administration.

The World Health Organization and Bangladeshi public health authorities strongly recommend exclusive breast-feeding for the first six-months of life to promote optimal growth, development, and health, and reduce infant mortality from common childhood diseases such as diarrhea and pneumonia [30]. Thus, it is important to ensure that oral administration of probiotics to infants did not negatively affect breastfeeding rates. No evidence was found that oral administration of probiotics decreased breastfeeding, preserving this important health practice.

There had been concern that underlying dysbiosis or impaired intestinal integrity might render infants susceptible to microbial translocation of the gut and infection by probiotic strains [31–33]. In this study no evidence of infection by probiotic strains was found; no illnesses were attributable to the probiotics. Although two investigations of probiotics in children in Bangladesh previously demonstrated safety, the children studied were older than those of our research [34, 35]. However, early gut microbiome assembly may be important in preventing dysbiosis; thus, testing probiotics in younger infants was imperative [36–40].

There have been some studies that suggest multistrain or multispecies probiotics may improve colonization or efficacy over monostrain probiotics [41, 42]; therefore, two probiotics were selected to test in combination in this study. While the selected probiotics have been well-studied for safety and efficacy in other situations, we cannot say that this choice of probiotics is optimal for infant health in Bangladesh. We are currently assessing duration of infant colonization and any physiologic signal of benefit in anticipation of a larger phase II-III trial. Other limitations of this study include a dropout rate of 29 % and the lack of blinding in the control arm, although this is not imperative in a phase I trial.

## Conclusions

In conclusion, this study found that a *L. reuteri* DSM 17938 and *B. longum* subspecies *infantis* 35624 combination, even given daily, is safe and well-tolerated in very young infants in Bangladesh. The confirmation of safety and acceptability of these probiotics in this study population lays the groundwork for investigation of the efficacy of these probiotics in improving the health of Bangladeshi infants.

## Abbreviations

ICDDR,B: International center for diarrheal disease research, Bangladesh
DSMB: Data safety monitoring board
